# Evaluating batch correction methods for image-based cell profiling

**DOI:** 10.1101/2023.09.15.558001

**Published:** 2023-09-17

**Authors:** John Arevalo, Robert van Dijk, Anne E. Carpenter, Shantanu Singh

**Affiliations:** Imaging Platform, Broad Institute of MIT and Harvard, Cambridge, Massachusetts, USA

**Keywords:** Batch correction, machine learning, image-based profiling, morphological profiling, Cell Painting, high-throughput phenotypic screening, Sphering, Harmony, Bulk analysis

## Abstract

High-throughput image-based profiling platforms are powerful technologies capable of collecting data from billions of cells exposed to thousands perturbations in a time- and cost-effective manner. Therefore, image-based profiling data has been increasingly used for diverse biological applications, such as predicting drug mechanism of action or gene function. However, batch effects pose severe limitations to community-wide efforts to integrate and interpret image-based profiling data collected across different laboratories and equipment. To address this problem, we evaluated seven top-ranked batch correction strategies for mRNA profiles in the context of a newly released Cell Painting dataset, the largest publicly accessible image-based dataset. We focused on five different use scenarios with varying complexity, and found that Harmony, a nonlinear method, consistently outperformed the other tested methods. Furthermore, we provide a framework, benchmark, and metrics for the future assessment of new batch correction methods. Overall, this work paves the way for improvements that allow the community to make best use of public Cell Painting data for scientific discovery.

## Introduction

Image analysis has become a cornerstone of biological and biomedical research. The use of fluorescence labeling combined with advanced optical microscopy now enables visualizing biological morphology, structures and processes at unprecedented spatial and temporal resolution. Furthermore, high-throughput microscopy can now extract precise information about morphological changes caused by thousands of specific genetic or chemical perturbations. Analysis of the resulting image-based profiles can be used to deduce gene functions and disease mechanisms, as well as characterize mechanism and toxicity of potential therapeutics^1^. The most commonly used multiplex image-based profiling assay is Cell Painting^2,3^. Cell Painting uses six dyes that label eight cellular components (nucleus, nucleolus, endoplasmic reticulum (ER), Golgi, mitochondria, plasma membrane, cytoplasm, and cytoskeleton) that are imaged in five channels. Thus, each image-based profile captures rich morphological features that are extracted using automatic image processing and analysis pipelines. The approach offers single-cell resolution, captures valuable population heterogeneity and provides information distinct from mRNA profiling ^4–9^ and protein profiling ^10^ at a cost lower than even the least expensive high-throughput bulk mRNA technique ^11^. Importantly, Cell Painting image-based profiles of cells exposed to different genetic or chemical perturbations have been successfully combined with machine learning strategies to generate predictive models that support key steps in drug discovery and development ^1^.

The broad applicability and the predictive power of Cell Painting data depends on having access to a large number of image-based profiles that can be used to either generate mechanistic hypotheses or build predictive models. Individual companies have begun to create proprietary datasets, but a large public Cell Painting dataset has been lacking, leaving the field unable to benefit from shared data as has been so crucial in other fields of biology such as genomics.

Thus, to enable construction of such a database, we recently partnered with colleagues from pharmaceutical companies, technology providers, and non-profit organizations to form the Joint Undertaking for Morphological Profiling (JUMP) Cell Painting Consortium^12^. These efforts resulted in the 2023 release of the first large-scale public dataset of image-based Cell Painting profiles, capturing data from more than 140,000 chemical and genetic perturbations (https://github.com/broadinstitute/cellpainting-gallery). A large, public dataset is most useful if it can be successfully queried using new profiles generated by individual laboratories in the future. This requires the community to have developed methods for aligning data generated with technical variation. With this in mind, the Consortium went to great effort to exchange compounds and generate images across twelve different laboratories with various equipment, enabling the development and evaluation of batch correction methods that could then be used by future data generators.

In general, batch effects arise from technical, non-biological factors that are commonly encountered during large-scale data collection efforts involving multiple sites. In the context of JUMP Cell Painting Consortium, technical factors (such as variations in the microscope used, microscope filters and settings, and cell growth conditions) all have effects on the image-based profiles we collected. Moreover, some batch effects occur even across batches from a single site, such as unintentional changes in lamp intensity, staining concentration, and cell seeding or growth rate. Additionally, the hierarchical structure of Cell Painting experiments, with each readout originating from a region in a well from a multi-well plate, which in turn comes from an experimental batch at a particular laboratory, further complicates the nature of the technical noise.

Only a handful of batch correction methods have been developed and tested for image-based profiling. No systematic and comprehensive comparison and evaluation of such methods has been performed, making it unclear whether the available methods offer a reliable approach for dealing with batch effects in image-based profiling. Such evaluations for single-cell RNA-seq (scRNA-seq) batch correction methods have revealed that no current method appears to outperform the others ^13,14^, and suggest that most of these methods should not be used without the guidance of an expert ^15,16^. Therefore, it remains unknown whether any of the scRNA-seq batch correction methods can be reliably applied to image-based profiles.

Here, we carried out a comprehensive analysis of seven batch correction methods. We used qualitative visualizations along with five metrics that capture reduction in batch effects, and eight metrics that capture preservation of biological signals. We used the newly released public database created by the JUMP Cell Painting Consortium ^12^ to test the performance in the context of five common use cases: multiple batches from a single laboratory, multiple laboratories using the same microscope with few and many compounds, and multiple laboratories using different microscopes with few and many compounds. Given the computational constraints of working with large image-based profiling datasets, we focused our evaluation on population-averaged well-level profiles rather than single-cell level profiles. We analyzed correction methods in the context of the replicate retrieval task (finding the replicate sample of a given compound across batches/laboratories), and we found that existing methods are effective in reducing batch effects in image-based profiles. Among the methods tested, Harmony, a non-linear method developed for processing scRNA-seq data, offered the best balance of batch removal and conservation of biological variance metrics. More broadly, the benchmark dataset, evaluation framework, and metrics we describe here will enable future assessment of novel batch correction methods as they emerge. This paves the way for improvements that allow the community to make best use of public Cell Painting data for scientific discovery.

## Results

### Selection of batch correction methods and evaluation strategies

A major part of our work was to comprehensively survey methods for batch correction, as well as strategies for their evaluation. Given the rapid advancements in the field of scRNA-Seq, particularly in the development of methods to address batch correction, we focused our attention on this area. We decided to test a subset of the better-performing methods identified in a recent analysis of scRNA-seq batch correction methods ^13,17^. These methods were available in Python and did not require additional preprocessing or metadata. Additionally, the chosen methods were representative of different approaches and included linear methods (Combat ^18^ and Sphering ^19^), neural-network based methods (scVI ^20^ and DESC ^21^), neighbor-based methods (Scanorama ^22^ and MNN ^23^), and a mixture-model based method (Harmony ^24^).

We will briefly summarize the main characteristics of these methods, to enable the reader to place our results in the appropriate context. Combat ^18^ models batch effects as multiplicative and additive noise to the biological signal and uses a Bayesian framework to fit linear models that factor such noise out of the readouts. Sphering^19^ computes a whitening transformation matrix ^25^ based on negative controls and applies this transformation to the entire dataset. It requires every batch to include negative control samples for which variations are expected to be solely technical. scVI ^20^ is a foundational model for representation learning in scRNA-seq data based on variational autoencoders. DESC ^21^ trains an autoencoder along with an iterative clustering algorithm to remove batch effects and preserve biological variation, and requires the knowledge of the biological variable of interest as input, which may be unknown at the batch correction stage. MNN ^23^ aligns representations between two batches by finding pairs of samples that are mutually nearest neighbors. Scanorama ^22^ computes mutual nearest neighbors across all of the batches and uses such neighbors as anchors to align the datasets. Harmony ^24^ is an iterative algorithm based on expectation-maximization that alternates between finding clusters with high diversity of batches, and computing mixture-based corrections within such clusters. All tested batch correction methods except Sphering require batch labels, Sphering alone requires negative control samples, and only DESC additionally requires biological labels. MNN, Scanorama, and Harmony necessitate recomputing batch correction across the entire dataset whenever new profiles are incorporated. While Sphering, Combat, scVI, and DESC don’t require recomputation, they don’t guarantee perfect corrections for new profiles from an unseen source.

Although these methods were developed for scRNA-seq data, scRNAseq produces single-cell mRNA profiles that are similar in structure to single-cell morphological profiles from the Cell Painting assay. Although the two profile types have inherent biological and statistical differences, most of the foundational assumptions, such as metrics in the vector space revealing similarities between them, are reasonable for the image-based profiling domain. However, we note a crucial difference in the manner we have applied these methods. Given the sheer volume of data in large image-based profiling datasets, which may contain billions of single cells compared to the millions typically found in scRNA-seq, it is computationally impractical to apply these methods at the single-cell level. Importantly three out of the seven methods require computing batch correction across the entire dataset. This means that subsampling, a strategy often used to manage large datasets, is not a viable option for those. Thus, we evaluated these methods based on their ability to correct batch effects in population-averaged well-level (or “pseudo-bulk”) profiles, rather than single-cell level profiles. This shift does alter the distribution of the features, but we believe this is an acceptable trade-off given the computational constraints and the overall goal of correcting batch effects at a broader level. Particularly for scVI, it’s worth noting that while the method assumes a negative binomial distribution for each feature—an assumption not met in image-based profiling datasets—we still included it in our evaluation. This decision was driven by scVI’s demonstrated high performance in scRNA-Seq^17^, and our interest in assessing its robustness when distributional assumptions are not fulfilled.

Beyond batch correction methods for scRNA-seq, we also reviewed those specifically designed for image-based profiling data analysis. A small handful of past research in this domain incorporated a step for batch correction. Such approaches can be split broadly into two categories: those based on (a) pre-computed feature transformation and (b) representation learning.

Pre-computed feature transformation approaches learn a transformation of features that have been extracted from images. In such workflows, common preprocessing/normalization steps have been described by Bray and Carpenter ^26^ to deal with interplate and intraplate normalization - these aim to reduce local variances, but they are limited when technical variations are strong. Sphering ^19^ is the most used batch correction method for feature-transformation-based profiles widely applied in Cell Painting pipelines ^27,28^ and was included in our testing.

Representation learning methods process the raw pixels from the fluorescent channel images, usually using neural-network-based approaches ^26–27,28^. Qian et al. ^29^ trained a generative adversarial network (GAN) to capture the batch effects from one batch using the style transfer framework. The GAN is then used to align the data by transferring the learned style to every image in the other batches. Despite their success, GANs face challenges during training related to convergence, mode collapsing and stability ^30^. More importantly, this approach requires selecting a reference batch, which may not be a trivial decision. Lin and Lu ^31^ addressed the batch effect problem differently, instead of proposing a separate method to correct the data they adapted the BatchNorm ^32^ strategy to learn a representation that accounts for batch information jointly with the downstream task. Because BatchNorm^32^ only works with differentiable neural networks, its scope is limited to gradient-based methods, and it requires integrating the downstream task into the loss function, which may differ depending on the question at hand, precluding correcting the data once for all possible applications of the data. In a similar spirit, Haslum et al. ^33^ proposed a modification for the teacher-student framework using self-supervised learning models such as DINO ^34^ and BYOL ^35^ that accounts for batch effects, reporting the kBET metric and qualitative visualizations to compare performance. Note that such models require data augmentation, which is not always feasible, especially for tabular information. Sypetkowski et al. ^36^ designed a neural network architecture including learning-based normalization and gradient reversal layers to solve the compound classification as a pretext task to correct for batch effects. Sypetkowski’s approach requires the training of a machine learning model to evaluate the performance, which limits the comparison with other methods. Overall, representation-learning methods for batch correction that require additional annotations as a way of supervision (e.g.^37^) are not applicable to all datasets, nor do they create a dataset useful for all applications. We therefore did not evaluate these methods in our study.

Next, we considered which dataset to use as a benchmark in our evaluation. RxRx1 ^36^ and RxRx3 ^38^ datasets are resources for image-based profiling, with millions of images associated with thousands of compounds, but derive from a single, highly-quality-controlled laboratory so cannot be used to assess methods aiming to correct more dramatic batch effects. We therefore chose to use the JUMP Cell Painting Consortium data as the only large public dataset that originated from a wide range of laboratories and from a range of instruments, thus capturing diverse batch effects.

Finally, we surveyed various evaluation strategies and considered them for the context of image-based profiling batch correction. In contrast to the RNA-seq domain where multiple benchmark studies exist ^13,17^, image-based profiling lacks a comprehensive comparison of batch effect correction methods, and there are no agreed evaluation criteria.

Evaluation techniques are typically split into qualitative and quantitative categories. Qualitative evaluations based on dimensionality reduction methods such as PCA, UMAP ^39^, t-SNE ^40^, or MDE ^41^ can expose clusters of biological labels, however it strongly compresses high dimensional data in a 2D space. This can be sensitive to hyper parameters and prone to subjectivity.

Quantitative evaluation of batch correction methods broadly take two forms: using bespoke metrics to directly quantify batch effect removal and conservation of biological variance, or indirectly examining the expressivity of corrected image-based profiles through subsequent analysis tasks. An indirect approach is to utilize downstream tasks such as image classification ^42^ and perturbation replicate retrieval across batches ^43^ as a measure of the expressivity of the image-based profiles after correction. Used in isolation, this indirect approach may fail to detect the presence of batch effects in situations where the experimental covariates (e.g. plate ID) correlate with the labels of the downstream task (e.g. samples’ replicates).

Sypetkowski et al. ^36^ propose a direct approach with their batch generalization and batch classification accuracy metrics. Batch generalization utilizes a perturbation classifier to compute the performance ratio between batches used for training the classifier and a held-out set of batches. A high ratio indicates a corrected representation that’s not heavily influenced by the batch. Batch accuracy trains a classifier to predict the batch ID based on the corrected representation; batch accuracy is low if batch correction has performed well. However, this approach also requires the tuning of additional classification pipelines during evaluation, making the comparison of batch correction methods somewhat less straightforward. Lastly, Kim et al.’s ^44^ evaluation provides another direct approach, using silhouette score, graph connectivity, and local inverse Simpson’s index (LISI) to quantify batch effects across different representation learning methods. Their evaluation is similar to our Scenarios 1 and 2, described below.

Our evaluation strategy differs from prior evaluations in image-based profiling literature in the following: 1) We evaluated 13 metrics, including those measuring conservation of biological variance and batch effect removal ^17^. 2) We evaluate more diverse scenarios (multiple microscopes, multiple laboratories, few/multiple perturbations, few/multiple replicates).

Having selected seven methods and 13 evaluation metrics in five scenarios, we then proceeded to our analysis.

### Scenario 1: Single microscope type, single laboratory, multiple batches, few compounds, multiple replicates

In this scenario we analyzed 306 “landmark” compounds present on the *Target2* plates (see Dataset description), one of which is present in each of the 13 experimental runs/batches produced by a single laboratory (source_6), a subset of the JUMP Cell Painting Consortium dataset. There were a median of 21 replicates per compound. Given that profiles were generated in the same laboratory, with many replicates and relatively low technical variance, this simplified scenario helped us establish a baseline for the best possible results while also guiding the preprocessing pipeline that could be applied across all more complex scenarios.

**Figure 2:**
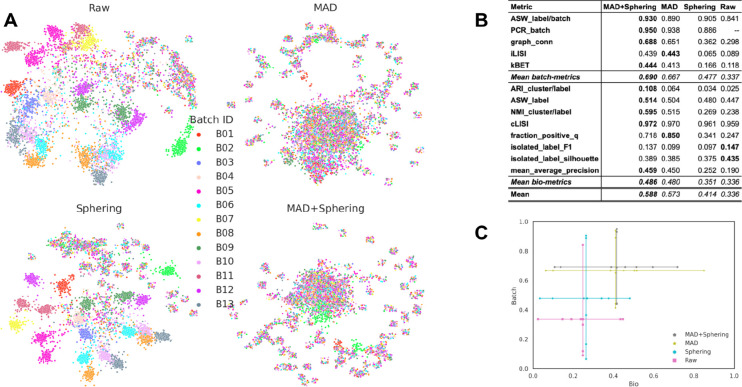
Preprocessing pipeline evaluation. **(A)** Qualitative comparison of normalization preprocessing options. Each color represents an experimental batch/run. Raw: Original morphological features. MAD: features normalized with MAD. Sphering: features normalized with Sphering. MAD+Sphering: Features after MAD and Sphering transformation, in that order. **(B)** Quantitative evaluation of the preprocessing steps on single-source data across 13 batches in Scenario 1. Columns represent each version of the data, with top rows for batch removal metrics and bottom rows for bio-metrics. The final row computes average scores. The PCR_batch metric compares the variance explained by the batch variable before and after correction, thus it can not be computed for raw representation as it is the original data. **(C)** Visualization of quantitative results post preprocessing in Scenario 1. The X-axis presents bio-metrics, the Y-axis displays batch removal metrics, and each cross represents a method’s performance, with intersections marking the average of the corresponding axis. Metrics with a low coefficient of variation (<0.01) across methods are omitted from this plot.

Image-based profiling requires carefully designing feature extraction and preprocessing pipelines to accurately represent the biological content of images. In particular, normalization is essential to cope with variability across features, in terms of their natural scales and covariances. Thus, we first examined the influence of normalization on the batch effects. We applied a sequence of two commonly used normalization steps (1) median absolute deviation (MAD) normalization ^45^, which rescales individual features; and (2) Sphering ^19^, which rescales all features in a multivariate manner. We applied MAD normalization per plate, and Sphering across the whole experiment. In both steps, the data was normalized against the negative controls which serve as the reference. In 2D projection of the raw profiles, i.e. profiles not transformed in any way, we saw that profiles are heavily influenced by the batch variation (Figure 2.A). Similarly, profiles subject to Sphering normalization alone also showed strong batch effects. By contrast, MAD normalization helped to mix the batches better, with or without Sphering, and appeared to retain information about areas of space filled with samples with distinctive phenotypes. Although informative, these qualitative visualizations were not sufficient to establish whether applying both transformations performed better than just MAD normalization alone. Thus, we performed a more quantitative comparison.

To quantify batch correction methods, we examined two kinds of metrics ^13,17^: (1) *batch removal* metrics that capture how well a method removes confounding variation from data; and (2) conservation of biological variance, termed here as *bio-metrics* for short, that capture the ability to preserve the variation related to a known biological variable (*e.g.* chemical compound in our case).

There is a trade-off between bio-metrics and batch removal metrics. A method could remove all of the confounding batch variation but simultaneously destroy the biological signal in the data. Also notice that some of these metrics are sensitive to the number of samples per concept of interest (e.g. compound), others focus on a notion of local neighborhood, and others consider more global arrangement. Because different metrics capture different aspects of the correction procedure, and no individual metric captures both effects, we took them all into consideration when making comparisons between multiple batch correction methods. The average of such metric scores has been shown to be a reasonable ranking criterion ^13,17^. To make interpretation easier, every metric here was normalized between 0 and 1, with 0 being the worst possible performance and 1 the perfect performance. These quantitative metrics (Figure 2.B) demonstrate that MAD+Sphering achieves the best performance in 4 out of 5 metrics measuring the effectiveness of batch removal, and 4 out of 8 bio-metrics measuring how well it preserves biological information. Overall, these results indicate that there is an advantage of applying these two preprocessing steps.

We also compared the methods by displaying the metrics in a cartesian plane (Figure 2.C). Again, we observed that applying both preprocessing steps was preferable according to most metrics. We therefore selected MAD + Sphering as our *Baseline* approach, in agreement with established protocols in the field ^44,45^. All remaining results in this paper apply this two-step preprocessing.

**Figure 3:**
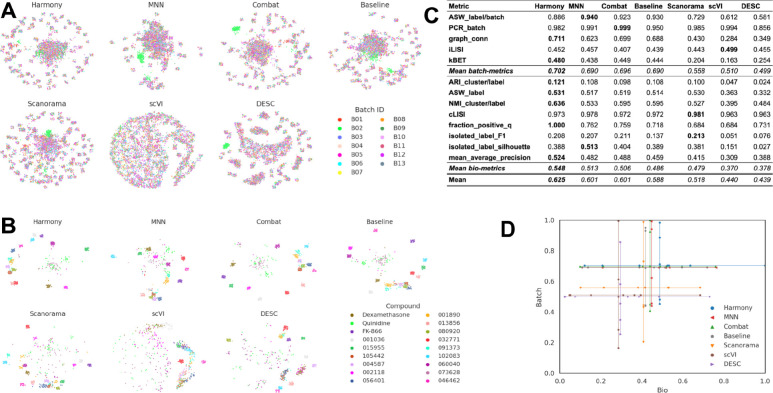
Evaluation Scenario 1. **(A)** Visualization of integrated data from multiple labs using seven batch correction methods. The experimental batch is represented by color, with the left-to-right, top-to-bottom layout reflecting the methods’ descending order of performance. **(B)** Same as (A) but showing a subset of image-based profiles, colored by compound. We selected 18 out of 306 compounds with replicates in different well positions to account for position effects that may cause profiles to look similar. Alphanumeric IDs denote positive controls. **(C)** Quantitative comparison of batch correction methods. Each column represents a method. The layout of the table mirrors Figure 2.B **(D)** Visualization of quantitative results after batch correction, mirroring Figure 2.C.

Following the preprocessing, we applied the following batch correction methods that have previously been identified as top-performing methods when applied to scRNAseq data: scVI ^20^, DESC ^21^, MNN ^23^, Scanorama ^22^, Combat ^18^, and Harmony ^24^. We reiterate that we used pseudo-bulk profiles and did not attempt batch correction at the single-cell level due to the computational time required to process up to billions of cells included in a typical image-based profiling experiment. Qualitatively, the 2D projection of the profiles after applying six different methods, in addition to our Baseline, shows no clusters associated with any particular batch (Figure 3.A), except for an outlier plate from Batch 2 that we identified during the course of this study (see *Dataset description*). This suggests that all the methods were successfully mixing the profiles from different batches as compared to the raw profiles (Figure 2.A). However, when visualizing the embeddings (*i.e.* 2D projections of corrected profiles), we noticed that Harmony and Combat were better at grouping the data points associated with the same compound than others. This suggests that such methods are more effective at preserving biological information (Figure 3.B). Here, positive control compounds exhibited better clustering (see Data Description), which was expected as these compounds were chosen based on the strong phenotype in previous Cell Painting experiments ^46^.

Based on the quantitative metrics (Figure 3.C), Combat, MNN and Harmony improved the performance with respect to the Baseline (MAD+Sphering) for both batch removal and bio-metrics criteria, with Combat slightly outperforming the other two. Surprisingly, Scanorama, DESC and scVI performed worse than the Baseline. The gap between the performance of different methods was also seen in the cartesian plot (Figure 3.D), where Harmony, MNN, Combat, and the Baseline achieve similar performance, while the remaining three methods are underperforming.

In summary, Scenario 1 helped us optimize our evaluation pipeline, showing that MAD + Sphering preprocessing produced higher quality data for subsequent batch effect correction. Among the seven batch correction methods tested, Combat, MNN, and Harmony performed best, while neural network-based methods (DESC and scVI) underperformed, perhaps due to fewer samples compared to typical scRNAseq datasets where millions of single cells are used to train the neural network. Further, scVI’s underperformance might be also attributed to its assumptions about data distribution, specifically the negative binomial distribution for each feature, which are not met in these data.

### Scenario 2: Single microscope type, multiple laboratories, few compounds, multiple replicates

**Figure 4.**
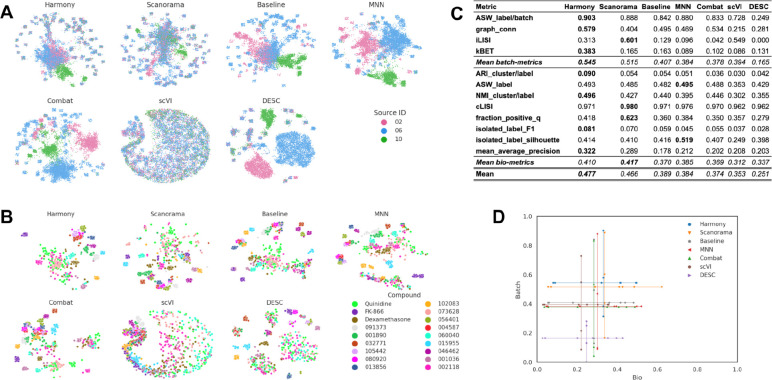
Evaluation Scenario 2. **(A)** Qualitative visualization of data from multiple laboratories integrated using seven batch correction methods. Each color represents the laboratory source. Left-to-right, top-to-bottom layout is given by methods’ quantitative performance in descending order. **(B)** Qualitative visualization of data from multiple laboratories integrated with seven batch correction methods. Compounds, colors, and layout order are as in Figure 3.B. **(C)** Performance of batch correction methods using batch removal metrics (Top) and conservation of biological variance metrics (Bottom). Methods order from left to right according to the mean score. **(D)** Performance comparison along batch removal and conservation of biological variance criteria.

In this scenario we analyzed the *Target2* 306 compounds from 43 experimental run/batches produced by three laboratories using the same model of microscope. We considered laboratory ID (identifier) as the variable of interest to be factored out because it is the most dominant confounding source (Figure 4.A). The Baseline approach was not able to integrate data from multiple laboratories, and batch effects were notable in the embedding, with clusters dominated by the confounding variable.

None of the methods completely eliminated the confounding variable effect as effectively as they did in Scenario 1 (Figure 4.A). However, we observed differences in their effectiveness, with Harmony and Scanorama clustering samples from multiple laboratories more effectively than other methods. On the other hand, the scVI’s embeddings resulted in the largest loss of information during the correction procedure. MNN and Combat did not differ significantly from the Baseline representation, while DESC groups paired sources into large blobs, reducing the structure of the embedding. When observing the embeddings labeled by compound (Figure 4.B), Harmony and Scanorama were able to group samples from the same compound. On the other hand, Combat and MNN showed clusters with Laboratory ID as the major separation criteria (see Figure 4.A), creating one cluster per laboratory–compound pair.

Consistent with these qualitative observations, Harmony was also the top performer in the quantitative metrics (Figure 4.C) for both batch removal and conservation of biological variance criteria. This result is consistent with Scenario 1 where Harmony achieved competitive performance. Taken together, we observed that by introducing stronger technical variations, i.e., analyzing data from multiple laboratories, the performance of batch effect correction methods decreased; however, methods’ rankings remained relatively consistent (Figure 4.D), with Harmony showing superior performance.

### Scenario 3: Single microscope type, multiple laboratories, multiple compounds, few replicates

**Figure 5.**
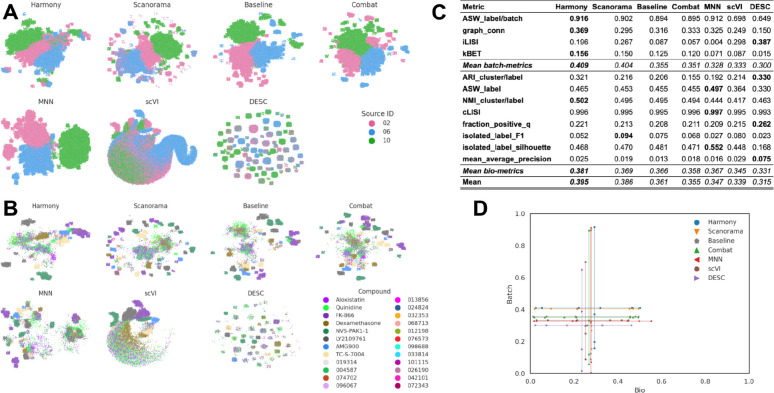
Evaluation Scenario 3. All panels mirror Figure 4, except **(B)** where the compounds were selected based on two criteria: we selected all eight positive controls and the top 16 compounds with the smallest dispersion amongst their replicates (measured by the median pairwise Euclidean distance among replicates).

In this scenario we analyzed 82,278 compounds from 43 experimental batches produced by three laboratories using the same model of microscope. We again factored out laboratory ID as the batch-related variable of interest because it is the most dominant confounding source (Figure 5.A). This scenario posed an additional challenge due to the reduced number of replicates for most of the compounds; around 16,000 compounds had only one replicate, and ~77,000 had 3 or fewer replicates. Importantly, the eight positive controls had around 2,500 replicates each.

We again began with our qualitative evaluation, which in this scenario was limited due to the large number of compounds to display. In general, several batch correction methods partially aligned the data, obtaining better clusters for compounds with many replicates, as expected (Figure 5.B). Quantitatively, Harmony and Scanorama again obtained better results than the Baseline, while Combat, MNN, scVI, and DESC underperformed in both bio-metrics and batch metrics (Fig Scenario3.C). As in Scenario 1, we attribute the low performance of scVI and DESC to the few available replicates per compound and unmet assumptions about data distribution. Overall, the increased complexity of the dataset resulted in a decreased gap across the methods (Figure 5.D). In other words, all methods struggled to remove the batch effects, as they remained notable after attempts at correction. Compared to Scenario 2, the methods were generally less effective when dealing with more compounds and fewer replicates.

### Scenario 4: Multiple microscope types, multiple laboratories, few compounds, multiple replicates

**Figure 6.**
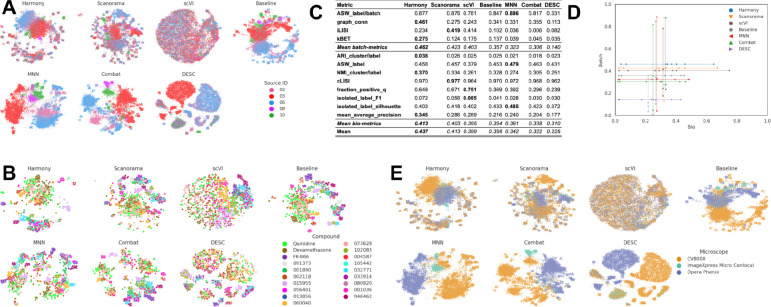
Evaluation Scenario 4. All panels mirror Figure 4, except **(E)** which is identical to **(A)** with the colors indicating microscope.

In this scenario we analyzed the *Target2* 306 compounds from 46 experimental run/batches produced by five laboratories using three different high-throughput imaging systems. Three sources used the CellVoyager CV8000 system, one source used the ImageXpress Micro Confocal system, and one source used the Opera Phenix system. This Scenario was similar to Scenario 2 given the same number of *unique* compounds; however, in Scenario 4 the batch effects are mainly influenced by the differences in imaging technologies (Figure. 6.E) We again considered laboratory ID as the technical variable of interest to be factored out to stay consistent with Scenarios 2 and 3.

We observed that Harmony and Scanorama were qualitatively better (Figure 6.A, 6.B), with Harmony generating the best quantitative results (Figure 6.C, 6.D). For MNN and Combat, their assumptions of orthogonality between biological signal and batch variation (MNN), and the additivity and linearity of batch effects (Combat), might not have been sufficient or did not hold true for this complex scenario, resulting in suboptimal performance. DESC also underperformed with respect to the Baseline, while scVI was slightly better. Compared to Scenario 2, the performance consistently decreased across all of the methods in most of the metrics, and the batch metrics exhibited the highest drop, indicating that differences in instrumentation had a strong impact on ability to align data.

### Scenario 5: Multiple microscope types, multiple laboratories, multiple compounds, few replicates

**Figure 7.**
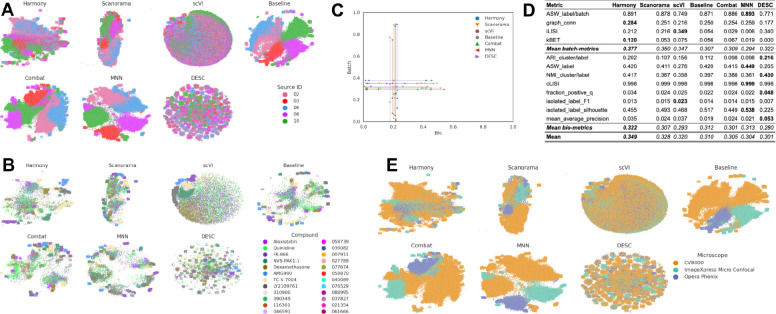
Evaluation Scenario 5. All panels mirror Figure 5, except **(E)** which is identical to **(A)** with the colors indicating microscope.

In the final, most complex scenario we analyzed 82,414 compounds from 60 experimental run/batches produced by five laboratories using different microscope systems as described in Scenario 4. Again, we found that the differences between microscopes was the strongest confounding factor (Figure 7.E). The 2D embeddings for DESC, Combat, MNN and Sphering form many clusters; however those are still dominated by the source (Figure 7.A). Qualitative comparison helped us to differentiate methods that failed to integrate data from different sources and/or laboratories, but was less useful to check whether they preserved the biological information (Figure 7.B). We attributed this not only to overplotting, but also to aggressive dimensionality reduction. For example, for the data corrected with Harmony, the first 10 principal components explained only 70% of the variance.

Harmony performed the best in terms of batch metrics and bio-metrics, and Scanorama was closely competitive. Although DESC scored best for several bio-metrics, it performed poorly in some of the batch metrics. This might be linked to the clustering observed in the 2D embeddings (Figure 7), where the local neighborhood was dominated by source; thus, even though the representation puts replicates of the same compound nearby, they are still separated by source.

## Discussion

High-throughput image-based assays represent powerful strategies for making biological discoveries and facilitating development of new therapeutics. These assays, such as Cell Painting, capture large amounts of data that can be used to connect genetic or pharmacological perturbations to specific changes in cellular morphology and/or phenotype. Over the last decade, the amount of image-based data has grown exponentially. However, the data benchmarking, processing, management, comparison and evaluation of image-based profiles remains challenging. One of the current challenges for the field has been the lack of robust batch correction methods, making it difficult to compare image-based profile data from different instruments, laboratories or even between different batches from the same laboratory. Without such solutions, public databases will be useless. Here, we address this problem by creating a framework for evaluation and systematic comparison of batch correction methods for image-based profiling. We applied our strategy to comparing seven different batch correction methods that have been originally developed for use with scRNAseq data. Similar to scRNAseq data, we found that no single metric is a complete indicator of performance and that surveying multiple metrics and qualitative visualizations improves our ability to compare the methods.

Overall, across five relevant scenarios of increasing complexity that we tested (batches within a lab, across laboratories and across imaging instrumentation, all with more or fewer compounds), we found that Harmony (and to a lesser extent, Scanorama), consistently outperforms the other tested methods. We consider Harmony to be a good trade-off: as a non-linear based approach it lacks the high complexity of the tested neural-network-based approaches, for which the available data in high-throughput experiments is often insufficient. In less complex scenarios, simpler methods may suffice; for example, for data generated in the same laboratory with many replicates of the compounds, the Baseline was sufficient to correct most of the batch effects, even though Harmony and Combat performed slightly better. However, we observed that as technical and biological variance increases, linear models such as Combat or Sphering fall short in aligning the data.

Although Harmony outperformed other methods tested, it’s important to point out a possible limitation for extensibility: it requires processing of all the data to re-align new batches against an existing dataset. This has a major impact on the ability of users to align their data with a large public dataset as it requires reprocessing and modifying existing representations. Therefore, further strategies, such as domain adaptation techniques ^47,48^ and self-supervised methods tailored for tabular data, ^49^ may represent promising alternatives. These methods, grounded in machine learning principles, are inherently flexible, allowing for incremental learning and the integration of new data without altering the previously established representations. While still in the exploratory stages for our context, these approaches have demonstrated potential in similar high-dimensionality scenarios and may provide an effective solution to batch alignment challenges that arise when incrementally expanding large datasets ^50,51^.

Importantly, we discovered that in the most difficult-to-align scenarios, none of the methods are able to adequately remove the batch effects. In fact, the best methods provide the greatest improvement in the least difficult-to-align scenarios. This raises a call for advancements from the field. Our study focused on bulk (population-averaged) profiles. It is possible that applying batch correction at the level of single cell profiles (likely subsampled), or even at a lower level using raw pixels, may yield better results. Among the methods we tested, Sphering, Combat, DESC, and scVI are suited for training on subsampled data at the single-cell level. Although neural networks have been explored, implementation and evaluation of these methods at the JUMP-dataset scale with billions of cells still represents a challenge.

## Experimental procedures

### Dataset description

The JUMP Cell Painting dataset ^12^ is a collection of several datasets that were either generated or reprocessed by the JUMP Cell Painting Consortium. The primary dataset (referred to as *cpg0016*) was generated during the data production phase of the JUMP-CP project. It comprises a compound dataset (116,753 perturbations), an Open Reading Frame gene overexpression dataset (15,142 perturbation) and a CRISPR gene knockout dataset (7,977 perturbations).

The compound dataset in *cpg0016* was generated across 11 data producing sites (or “sources”). Each source, apart from source_7, exchanged their nominated compounds with either two or four other sources where they were assayed using different instruments and microscopes (details are provided in the Methods section of ^12^). To simplify logistics, the sites were split into two groups; each site exchanged compounds only within their respective groups. In this paper, we use only a subset of the compound dataset, comprising all the 82,414 chemical perturbations from one of the two groups.

A positive control plate of 306 diverse compounds – named *JUMP-Target-2-Compound* – was run with every batch of data generation. These plates not only allow alignment of data within the JUMP dataset, but also with future datasets generated outside the consortium and can thus be considered as control plates. In this paper, *JUMP-Target-2-Compound* plates are referred to as *Target2* plates for brevity; the remaining plates – comprising the 116,753 chemical perturbations – are referred to as *Production* plates.

All Production plates have negative controls and several positive controls to identify and/or correct for different experimental artifacts.

- Dimethyl Sulfoxide (DMSO) -treated wells serve as *negative controls*. They are used for detecting and correcting plate to plate variations. They can also be used as a baseline for identifying perturbations with a detectable morphological signal.
- Using a pilot dataset (CPJUMP1 ^46^) generated by the JUMP Cell Painting Consortium, we identified a set of eight compounds with the most distinct signatures from each other and from DMSO; these are used as *positive controls*. The identity of each compound is provided in Supplementary Table 1 of ^12^.

Images in the study were processed using CellProfiler bioimage analysis software to measure cellular features such as intensity, granularity, texture, and shape. The image-based profiling involved processing the CellProfiler features using pycytominer ^52^ to normalize each feature and filter out redundant and invariant features. The available data includes images, single-cell profiles, and different levels of well-level profiles. Please refer to ^12^ for detailed information about the image and data processing and data availability.

As noted in *Results*, we observed an outlier plate during the course of our analysis: Images from plate 110000293093 in Batch 2 of source_6 had been reprocessed because it failed in the initial run. This plate likely contains a stronger technical deviation than the others due to the separate reprocessing event. We didn’t delve into investigating this anomaly further, as our focus remained on broader patterns of batch correction across the dataset.

### Metrics

We computed five metrics for batch removal efficiency and eight metrics to measure how well the correction preserves biological information, previously reported in single-cell RNA-Seq benchmarks ^13,17^. They are implemented in the scib package^1^. To make interpretation easier, every metric is normalized between 0 and 1 with 0 being the worst possible performance and 1 the perfect performance. A short description of each method is provided below. More details are provided in Luecken et al. ^17^.

- *ASW-based*: The average silhouette width (ASW) is a measure of how well a sample is assigned to its cluster. **ASW_label** metric considers the compound as the cluster ID; while **ASW_label/batch** metric considers the confounding variable as the cluster ID.
- **PCR_batch**: Computes the *R*^2^ between each principal component and the confounding variable before and after correction. The difference between the sum of such values before and after correction determines the batch removal efficiency. We reported PCR_batch in Scenario 1 using the *Raw* as representation before correction. We do not report PCR_batch in Scenario 2 onwards because the *Raw* representation does not provide an insightful comparison in more complex scenarios.
- **Graph_conn**: Uses the k-NN graph to measure the connectivity of each sample and those that belong to the same compound. This metric assumes If the batch effects were effectively removed, then the elements of the same biological concept should be close together.
- *LISI-based*: LISI is a metric based on the Simpson’s diversity Index to measure the diversity of a sample’s local neighborhood in the data. **iLISI** uses the confounding variable to measure such diversity; while **cLISI** uses the compound annotations to measure such diversity.
- **kBET:** Compares the global distribution and the local distribution of the confounding variable for each sample in the dataset. If the confounding variable is effectively removed, then such distributions should be similar.
- **ARI_cluster** *and* **NMI_cluster**: Adjusted Rand Index (ARI) and Normalized Mutual Information (NMI) measure the agreement between two clustering assignments. **ARI_cluster** and **NMI_cluster** metrics computes agreement between the clustering assignments of the Louvain algorithm applied over the corrected data and the compound annotations.
- *Isolated_label metrics*: **Isolated_label_F1** and **isolated_label_silhouette** metrics aim to capture the effectiveness of the correction methods in compounds not present in all of the batches by computing F1 and ASW scores within each cluster generated by the Louvain algorithm on the compounds present in the least number of batches.

We additionally report **mean average precision** as a bio-metric (how distinguishable are samples of the same compound from other compounds – i.e., are they retrieved towards the top of a list of samples ranked by similarity to the query compound?). We measure similarity between samples using cosine similarity.

Following the information retrieval convention, each sample in the dataset is considered a query; K other samples sharing the same compound are considered positive elements to be retrieved; and N samples belonging to the same query’s plate with different compound annotations are considered negative elements. The rank list is computed using the cosine similarity between the corrected profiles. Once computed, we estimate a p-value for each query by computing 10000 shuffled ranked lists mimicking the size K+P (random baseline), where K is the number of positive matches and P is the number of reference samples. Finally, we average the AP, termed **mean_average_precision**, of each compound and combine their p-values using the Fisher’s method. We computed a q-value for each compound by applying the Benjamini & Hochberg procedure to control for the false discovery rate. The **fraction_positive_q** metric is the ratio of compounds for which its q-value is lower than 0.05.

### Implementation

- MAD scales profile vectors feature-wise using mean absolute deviation $x=\frac{x\;-\;median\left(x\right)}{mad\left(x\right)+\in }$ at the plate level. We added an epsilon value ∈ to the denominator to avoid zero-division errors. $\in \sim 10^{U\left(-4,\;1\right)}$ and Sphering regularization $\lambda \sim 10^{U\left(-6,\;1\right)}$ hyperparameters were randomly explored and the best combination out of 25 runs was chosen according to the performance in the Target2 data.
- For scVI, we shifted the data to the feasible space. (i.e. transform each feature ${\hat{x}}_{i}=x_{i}-min\left(x\right)+1$)
- Metrics computed with the scib package are reported only for compounds with at least 5 replicates.
- The nature of the image-based profiles data involving low number of replicates and high number of compounds limits metrics such as kBET that rely on a higher (>15) number of samples per biological concept.
- mean_average_precision is the only metric able to capture the performance of the models when there are as few as only two replicates of a compound.
- We use Minimal Distortion Embedding (MDE) ^41^ for embedding visualization with default parameters from the scib package. MDE is a framework that generalizes PCA and UMAP dimensionality reduction methods. Scib implementation scales to large datasets and allows the usage of GPU for faster computation.

## Figures and Tables

**Figure 1:**
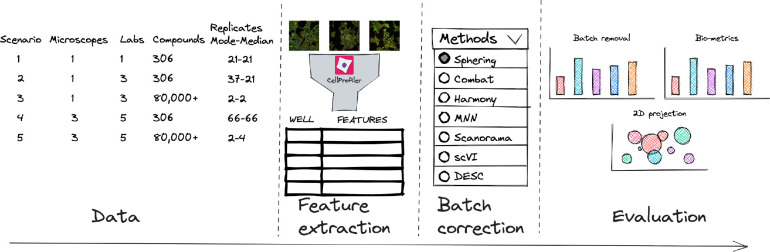
Evaluation pipeline. We evaluated five image-based profiling scenarios with different image acquisition equipment (high-throughput microscopes), laboratory, number of compounds and number of replicates. We preprocessed the readouts using state-of-the-art preprocessing pipeline for image analysis. We compared seven batch correction methods, and compared their performance using qualitative and quantitative metrics.

## Data Availability

All code to reproduce this analysis is located at https://github.com/carpenter-singh-lab/2023_Arevalo_BatchCorrection. All the corresponding data is available as part of the *cpg0016* dataset ^12^, available from the Cell Painting Gallery on the Registry of Open Data on AWS (https://registry.opendata.aws/cellpainting-gallery/).

## Footnotes

1
https://github.com/theislab/scib
