# Supplementary material for: Evaluating batch correction methods for image-based cell profiling
Source: bioRxiv. 2024 Feb 28:2023.09.15.558001. Originally published 2023 Sep 17. Preprint. [Version 3] doi: 10.1101/2023.09.15.558001 (PMC10516049; doi:10.1101/2023.09.15.558001)
Supplement: Supplement 1 [file NIHPP2023.09.15.558001v3-supplement-1.pdf]

bioRxiv preprint doi: https://doi.org/10.1101/2023.09.15.558001; this version posted February 28, 2024. The copyright holder for this preprint (which was not certified by peer review) is the author/funder, who has granted bioRxiv a license to display the preprint in perpetuity. It is made available under aCC-BY 4.0 International license.

Supplementary material

Preprocessing exploration

We explored combinations of the four steps above along with strategies to deal with outliers such as imputation (with KNN and median), clipping, and feature dropping. We choose the most convenient pipeline based on the mAP scores in Scenario 1. See https://github.com/carpenter-singh-lab/2023_Arevalo_BatchCorrection/issues/4 for further details about the exploration.

bioRxiv preprint doi: https://doi.org/10.1101/2023.09.15.558001; this version posted February 28, 2024. The copyright holder for this preprint (which was not certified by peer review) is the author/funder, who has granted bioRxiv a license to display the preprint in perpetuity. It is made available under aCC-BY 4.0 International license.

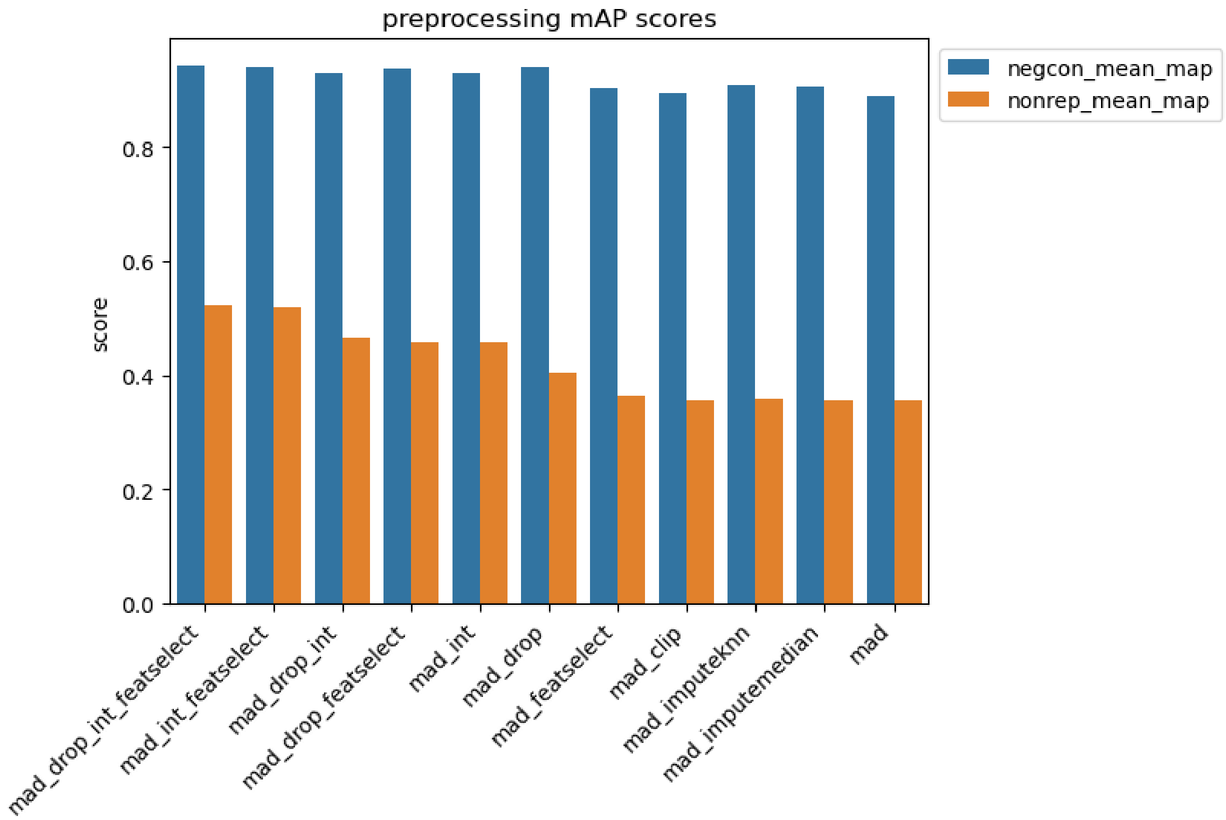

Supplementary Figure A preprocessing mAP scores

bioRxiv preprint doi: https://doi.org/10.1101/2023.09.15.558001; this version posted February 28, 2024. The copyright holder for this preprint (which was not certified by peer review) is the author/funder, who has granted bioRxiv a license to display the preprint in perpetuity. It is made available under aCC-BY 4.0 International license.

Scenario 1

A

Method	Batch correction: Graph connectivity	KBET	LISI batch	Silhouette batch	Bio metrics: LISI label	Leiden ARI	Leiden NMI	Silhouette label	mAP (controls)	mAP (nonrep)	Aggregate score: Batch correction	Bio metrics	Overall
**Harmony**	0.79	0.42	0.40	0.74	0.98	0.07	0.60	0.55	0.95	0.60	0.59	0.62	0.61
**Combat**	0.78	0.44	0.41	0.84	0.98	0.05	0.56	0.53	0.94	0.55	0.62	0.60	0.61
**MNN**	0.78	0.44	0.40	0.84	0.98	0.07	0.56	0.52	0.94	0.54	0.61	0.60	0.61
**DESC**	0.78	0.39	0.42	0.74	0.98	0.06	0.58	0.55	0.95	0.56	0.58	0.61	0.60
**Baseline**	0.77	0.41	0.39	0.85	0.98	0.06	0.55	0.52	0.94	0.52	0.61	0.60	0.60
**Sphering**	0.74	0.39	0.37	0.87	0.98	0.06	0.54	0.52	0.94	0.51	0.59	0.59	0.59
**Scanorama**	0.54	0.72	0.53	0.59	0.98	0.09	0.56	0.45	0.94	0.49	0.59	0.59	0.59
**scVI**	0.72	0.39	0.43	0.70	0.98	0.06	0.58	0.53	0.94	0.56	0.56	0.61	0.59

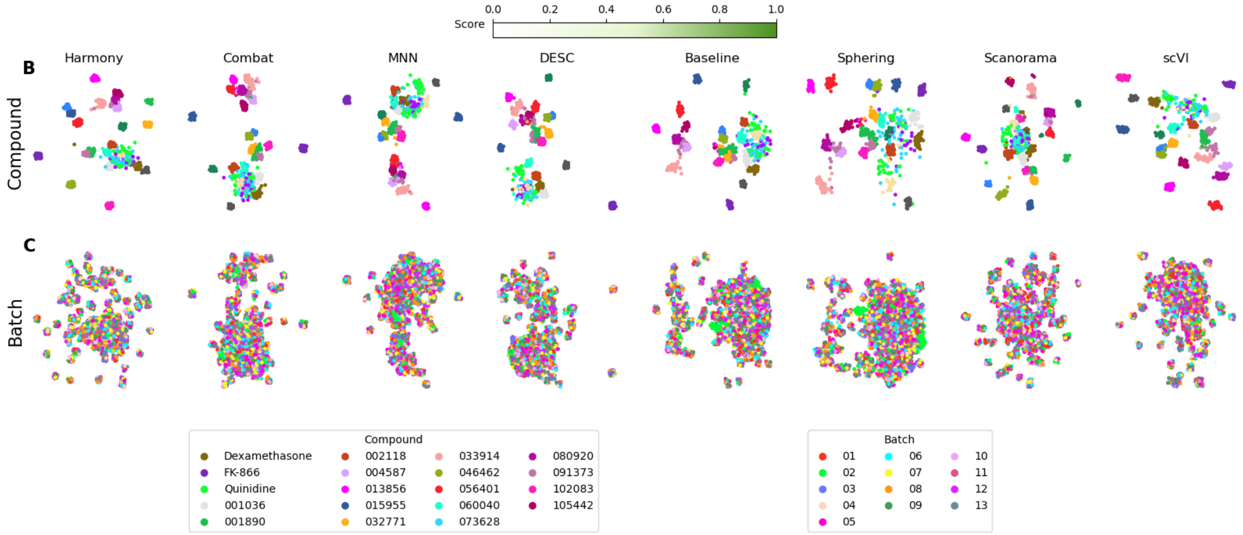

Supplementary Figure B. *Evaluation Scenario 1.* ***A)*** *Quantitative comparison of seven batch correction methods measuring batch effect removal (four batch correction metrics) and conservation of biological variance (six bio-metrics). Metrics are mean aggregated by category. Overall score is the weighted sum of aggregated batch correction and bio-metrics with 0.4 and 0.6 weights respectively. Visualization of integrated data colored by* ***B)*** *Compound, and* ***C)*** *Batch. Left-to-right layout reflects the methods' descending order of performance. We selected 18 out of 306 compounds with replicates in different well positions to account for position effects that may cause profiles to look similar. Alphanumeric IDs denote positive controls.*

bioRxiv preprint doi: https://doi.org/10.1101/2023.09.15.558001; this version posted February 28, 2024. The copyright holder for this preprint (which was not certified by peer review) is the author/funder, who has granted bioRxiv a license to display the preprint in perpetuity. It is made available under aCC-BY 4.0 International license.

Scenario 2

A

Method	Batch correction				Bio metrics						Aggregate score		
	Graph connectivity	KBET	LISI batch	Silhouette batch	LISI label	Leiden ARI	Leiden NMI	Silhouette label	mAP (controls)	mAP (nonrep)	Batch correction	Bio metrics	Overall
**Scanorama**	0.53	0.66	0.67	0.79	0.98	0.04	0.42	0.47	0.92	0.46	0.66	0.55	0.59
**Harmony**	0.64	0.26	0.55	0.86	0.98	0.04	0.42	0.48	0.92	0.46	0.58	0.55	0.56
**scVI**	0.59	0.16	0.48	0.74	0.98	0.04	0.45	0.47	0.90	0.47	0.49	0.55	0.53
**MNN**	0.62	0.10	0.19	0.76	0.98	0.04	0.42	0.48	0.90	0.42	0.42	0.54	0.49
**Combat**	0.62	0.05	0.16	0.76	0.98	0.03	0.42	0.48	0.91	0.42	0.40	0.54	0.48
**DESC**	0.59	0.11	0.32	0.67	0.97	0.03	0.38	0.42	0.89	0.42	0.42	0.52	0.48
**Baseline**	0.60	0.04	0.17	0.75	0.98	0.04	0.42	0.48	0.90	0.41	0.39	0.54	0.48
**Sphering**	0.58	0.03	0.13	0.76	0.98	0.03	0.40	0.48	0.91	0.39	0.38	0.53	0.47

Score 0.0 0.2 0.4 0.6 0.8 1.0

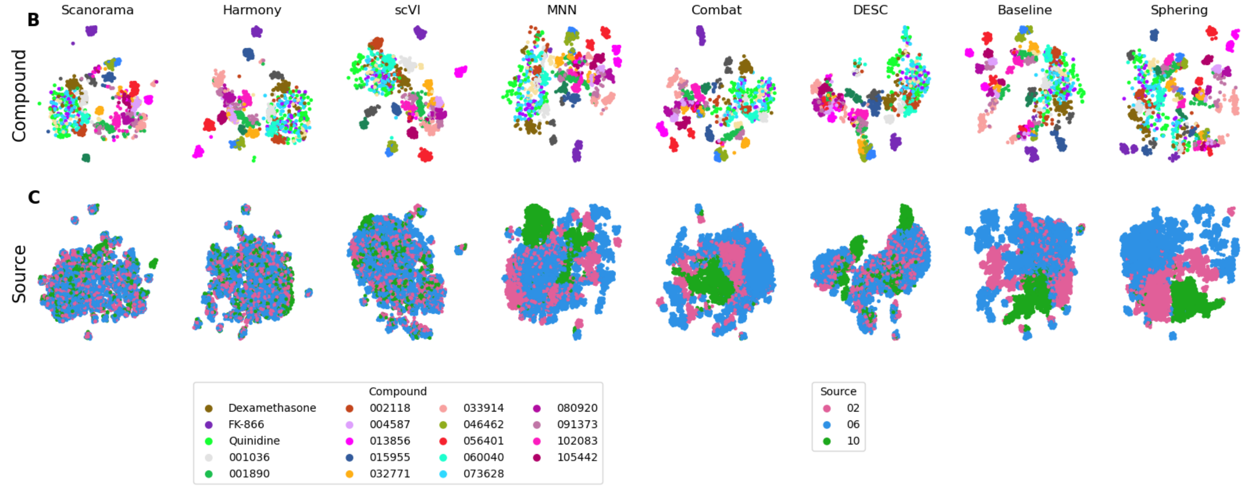

Supplementary Figure C. *Evaluation Scenario 2.* ***A)*** *Quantitative comparison of seven batch correction methods measuring batch effect removal (four batch correction metrics) and conservation of biological variance (six bio-metrics). Metrics are mean aggregated by category. Overall score is the weighted sum of aggregated batch correction and bio-metrics with 0.4 and 0.6 weights respectively. Visualization of integrated data colored by* ***B)*** *Compound, and* ***C)*** *Source. Left-to-right layout reflects the methods' descending order of performance. We selected 18 out of 306 compounds with replicates in different well positions to account for position effects that may cause profiles to look similar. Alphanumeric IDs denote positive controls.*

bioRxiv preprint doi: https://doi.org/10.1101/2023.09.15.558001; this version posted February 28, 2024. The copyright holder for this preprint (which was not certified by peer review) is the author/funder, who has granted bioRxiv a license to display the preprint in perpetuity. It is made available under aCC-BY 4.0 International license.

Scenario 3

A

	Batch correction				Bio metrics						Aggregate score		
**Method**	Graph connectivity	KBET	LISI batch	Silhouette batch	LISI label	Leiden ARI	Leiden NMI	Silhouette label	mAP (controls)	mAP (nonrep)	Batch correction	Bio metrics	Overall
**Scanorama**	0.54	0.33	0.36	0.76	1.00	0.01	0.29	0.27	0.20	0.05	0.50	0.30	0.38
**Harmony**	0.54	0.06	0.25	0.79	1.00	0.02	0.32	0.31	0.24	0.05	0.41	0.32	0.36
**scVI**	0.54	0.07	0.27	0.74	1.00	0.02	0.32	0.23	0.24	0.05	0.40	0.31	0.35
**Sphering**	0.54	0.02	0.00	0.80	1.00	0.00	0.26	0.35	0.21	0.03	0.34	0.31	0.32
**Combat**	0.54	0.03	0.02	0.76	1.00	0.01	0.29	0.30	0.22	0.04	0.34	0.31	0.32
**MNN**	0.54	0.03	0.01	0.75	1.00	0.01	0.29	0.29	0.20	0.04	0.33	0.30	0.32
**Baseline**	0.54	0.03	0.00	0.74	1.00	0.00	0.28	0.29	0.19	0.03	0.33	0.30	0.31
**DESC**	0.53	0.00	0.15	0.43	1.00	0.02	0.38	0.04	0.14	0.02	0.28	0.27	0.27

Score: 0.0 0.2 0.4 0.6 0.8 1.0

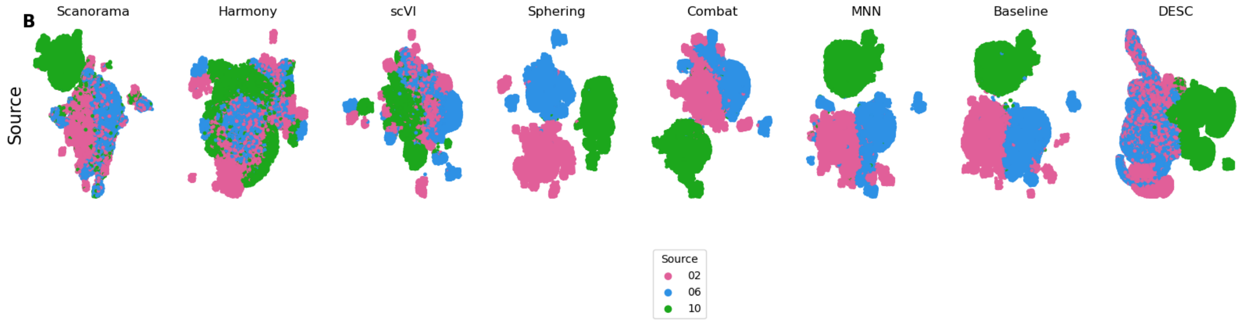

Supplementary Figure D. *Evaluation Scenario 3.* ***A)*** *Quantitative comparison of seven batch correction methods measuring batch effect removal (four batch correction metrics) and conservation of biological variance (six bio-metrics). Metrics are mean aggregated by category. Overall score is the weighted sum of aggregated batch correction and bio-metrics with 0.4 and 0.6 weights respectively.* ***B)*** *Visualization of integrated data colored by Source. Left-to-right layout reflects the methods' descending order of performance.*

bioRxiv preprint doi: https://doi.org/10.1101/2023.09.15.558001; this version posted February 28, 2024. The copyright holder for this preprint (which was not certified by peer review) is the author/funder, who has granted bioRxiv a license to display the preprint in perpetuity. It is made available under aCC-BY 4.0 International license.

Scenario 5

A

Method	Batch correction				Bio metrics						Aggregate score		
	Graph connectivity	KBET	LISI batch	Silhouette batch	LISI label	Leiden ARI	Leiden NMI	Silhouette label	mAP (controls)	mAP (nonrep)	Batch correction	Bio metrics	Overall
**Scanorama**	0.34	0.14	0.42	0.83	1.00	0.01	0.29	0.27	0.23	0.05	0.43	0.31	0.36
**Harmony**	0.34	0.03	0.25	0.84	1.00	0.02	0.28	0.32	0.26	0.05	0.36	0.32	0.34
**scVI**	0.34	0.04	0.23	0.77	1.00	0.01	0.28	0.25	0.24	0.05	0.34	0.30	0.32
**Sphering**	0.34	0.01	0.00	0.84	1.00	0.00	0.21	0.38	0.22	0.03	0.30	0.31	0.30
**Combat**	0.34	0.01	0.02	0.81	1.00	0.00	0.24	0.31	0.24	0.04	0.30	0.30	0.30
**MNN**	0.34	0.03	0.01	0.81	1.00	0.00	0.23	0.31	0.22	0.04	0.29	0.30	0.30
**Baseline**	0.34	0.01	0.01	0.80	1.00	0.00	0.23	0.31	0.21	0.03	0.29	0.30	0.29
**DESC**	0.33	0.01	0.27	0.58	1.00	0.02	0.34	0.01	0.22	0.04	0.30	0.27	0.28

Score: 0.0 0.2 0.4 0.6 0.8 1.0

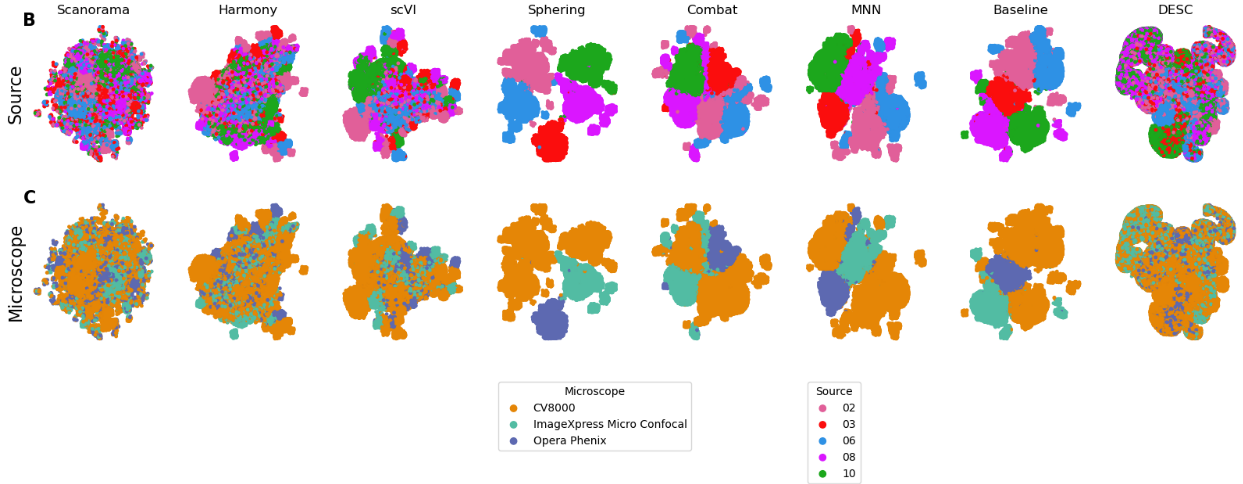

Supplementary Figure E. *Evaluation Scenario 5.* ***A)*** *Quantitative comparison of seven batch correction methods measuring batch effect removal (four batch correction metrics) and conservation of biological variance (six bio-metrics). Metrics are mean aggregated by category. Overall score is the weighted sum of aggregated batch correction and bio-metrics with 0.4 and 0.6 weights respectively. Visualization of integrated data colored by* ***B)*** *Source, and* ***C)*** *Microscope. Left-to-right layout reflects the methods' descending order of performance.*

Isolated compounds performance

Around 30% of the compounds of Scenario 3 are present in all three sources (sources 2, 6, and 10). We used this scenario to assess the replicate retrieval performance of sub-populations of compounds that are not shared between different batches (i.e. sources, in this setup). We used the corrected profiles from the best-performing correction method in the scenario – Harmony – to evaluate. We picked the 10,136 compounds that present in sources 2 and 6 but not in source 10 (i.e., they are isolated to sources 2 and 6). We compared the performance of this subpopulation (named as **two sources** in Sup Figure F) with the performance of a subpopulation of 23,782 compounds present in all of the three sources (named as **three sources** in Sup Figure F). Then we compute the mAP (control) score for each subpopulation,

bioRxiv preprint doi: https://doi.org/10.1101/2023.09.15.558001; this version posted February 28, 2024. The copyright holder for this preprint (which was not certified by peer review) is the author/funder, who has granted bioRxiv a license to display the preprint in perpetuity. It is made available under aCC-BY 4.0 International license.

noting that we pick only the replicates from source 2 and source 6 and ignoring the replicate from source 10. We observed that the compounds that exclusively belong to **two sources** performed better than compounds present in all **three sources**, which contradicts the over-correction hypothesis. A likely explanation is that the correction task gets more difficult as there are more sources to align.

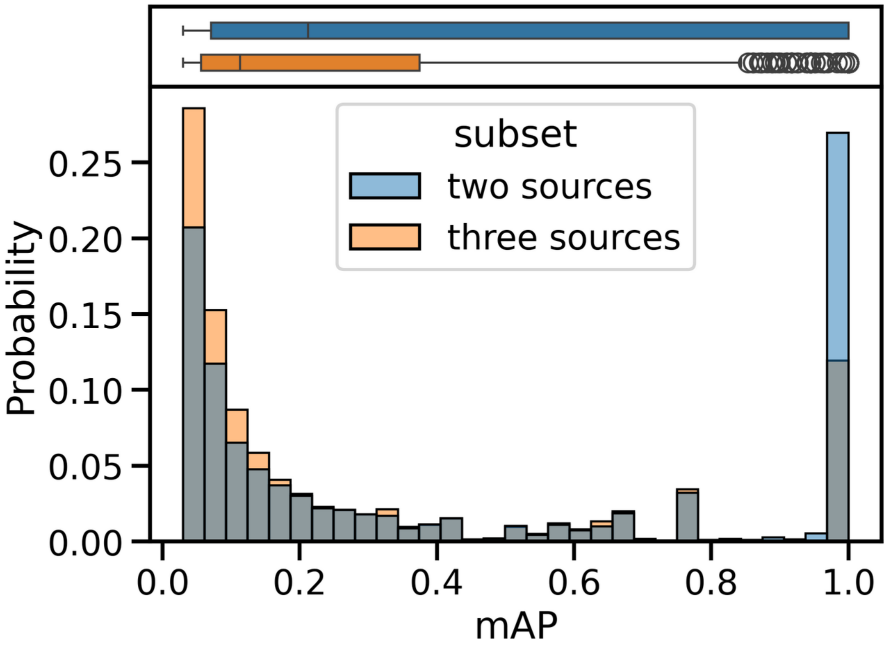

Supplementary Figure F: Comparison of the replicate retrieval performance (mAP) of sub-populations of compounds that are not shared between different batches. The sub-population present in only *two sources* performed better than the sub-population in *three sources.* Data extracted from the Scenario 3.

Runtime analysis

We measured the runtime for non-gpu methods and metrics across the five scenarios on a c6i.16xlarge AWS EC2 instance equipped with 64 cores and 128GB of RAM. A log-log plot of the results (Sup Figure G) reveals a linear-like trend, suggesting a power-law relationship between runtime and sample size. Extrapolating this trend, applying Harmony (a top-performant method) at the single-cell level (Sup Table A) would be prohibitively time-consuming: approximately 2.6 hours for a single plate, 33 hours for a single batch, and 11 days for a single source (there are 13 sources in the full JUMP Cell Painting dataset). Furthermore, loading such a source would require 2.7 TB of memory.

bioRxiv preprint doi: https://doi.org/10.1101/2023.09.15.558001; this version posted February 28, 2024. The copyright holder for this preprint (which was not certified by peer review) is the author/funder, who has granted bioRxiv a license to display the preprint in perpetuity. It is made available under aCC-BY 4.0 International license.

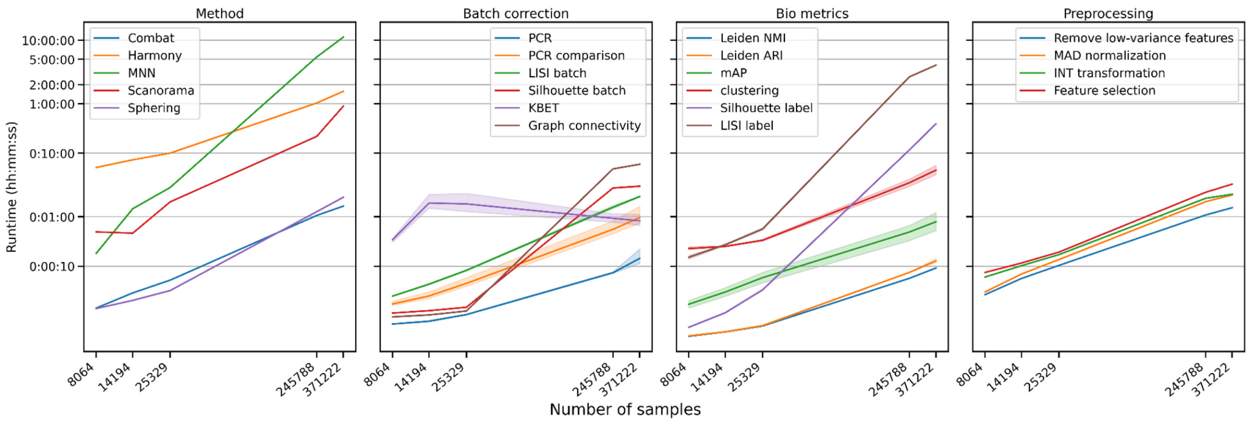

Supplementary Figure G: Runtime analysis of methods, metrics, and preprocessing steps for all the scenarios. Scenario 1, 2, and 4 (first three ticks in the x-axis) have *JUMP-Target-2-Compound* plates only with ~300 unique compounds. Scenarios 3 and 5 (last two ticks in the x-axis) have Production plates with ~80,000 unique compounds. Both axes are log-scaled. KBET runtime trend is constant because it only evaluates compounds with more than 15 replicates. The Clustering step is required for LISI, NMI, ARI, Graph connectivity, and KBET.

Level	Mean	Median
**Per Well**	1,846	1,520
**Per Plate**	708,819	575,946
**Per Batch**	14,129,906	11,292,977
**Per Source**	172,384,854	147,953,548
**Total (sources 2,3,6,8,10)**	861,924,272	
**Total (all 13 JUMP sources)**	1,834,731,584	

Supplementary Table A: Count of single cells at different levels in the JUMP CP Dataset.

bioRxiv preprint doi: https://doi.org/10.1101/2023.09.15.558001; this version posted February 28, 2024. The copyright holder for this preprint (which was not certified by peer review) is the author/funder, who has granted bioRxiv a license to display the preprint in perpetuity. It is made available under aCC-BY 4.0 International license.

Performance distribution

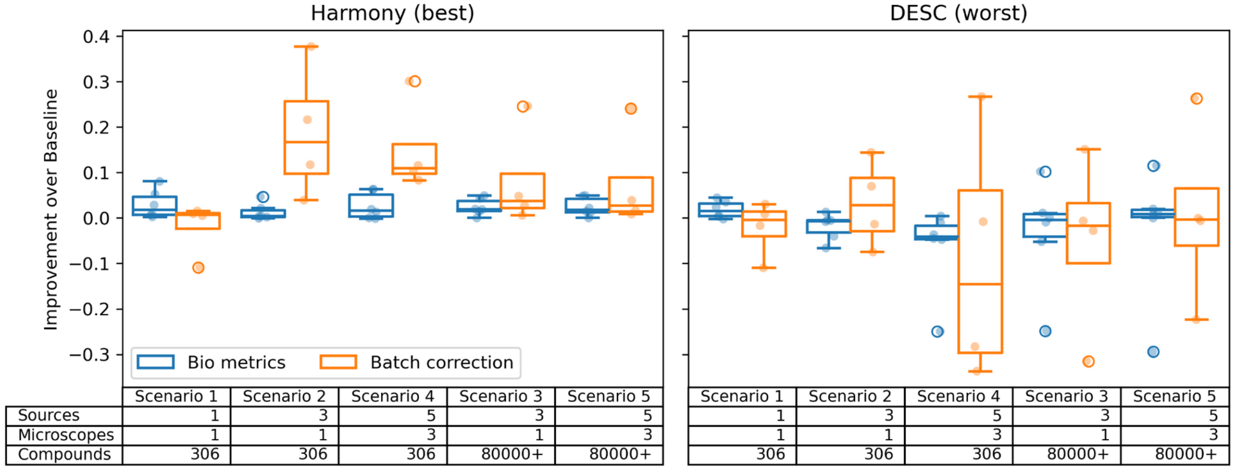

Supplementary Figure H: Comparison of best and worst batch correction methods, reflecting the variability of the performance with respect to the complexity of the scenarios (scenarios are sorted by overall mean score).

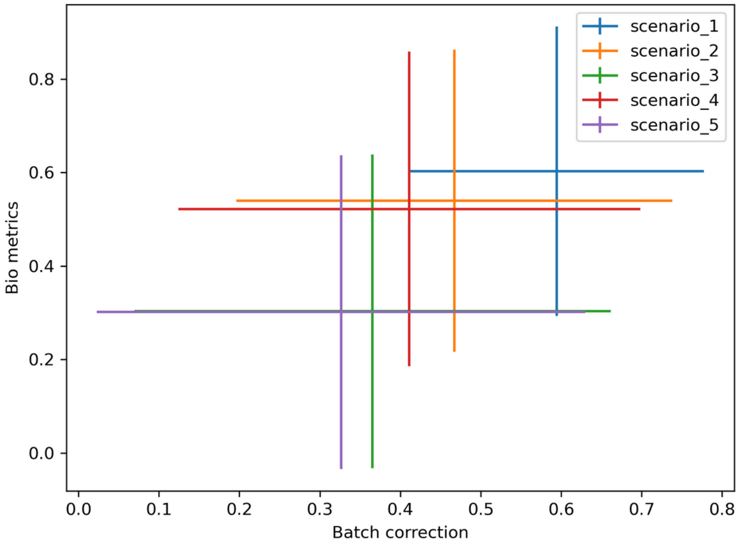

Supplementary Figure I: Scatter plot of the mean Batch correction and bio-metrics for all the tested methods across the five scenarios, reflecting the increasing difficulty of scenarios. Bars represent one standard deviation in the respective axis.

Implementation notes

- For scVI, we shifted the data to the feasible space. (i.e. transform each feature $\hat{x}_i = x_i - min(x) + 1$)
- The nature of the image-based profile data involving low number of replicates and high number of compounds limits kBET, which relies on a higher (>15) number of samples

bioRxiv preprint doi: https://doi.org/10.1101/2023.09.15.558001; this version posted February 28, 2024. The copyright holder for this preprint (which was not certified by peer review) is the author/funder, who has granted bioRxiv a license to display the preprint in perpetuity. It is made available under aCC-BY 4.0 International license.

per biological concept.

- mAP is the only metric able to capture the performance of the models when there are as few as only two replicates of a compound.
- We optimize the preprocessing pipeline based on a mAP.
- We adjust DESC convergence hyperparameters to avoid collapsed representations (default parameters converged to vectors with only -1, 1 values (output from a tanh activation))
- We increase the number of Harmony clusters from 50 to 300 and iterations from 10 to 20.
- We increase the number of latent dimensions in scVI from 10 to 30.
- Combat implementation from scanpy has no hyperparameters.
- We use the default hyperparameters for MNN (neighbor size=20), Scanorama (KNN=20, alpha=0.1, sigma=15) and scVI (num_units=128, dropout=0.1).
